# Phenomic Imaging

**DOI:** 10.1007/s43657-023-00128-8

**Published:** 2023-11-03

**Authors:** Lizhen Lan, Kai Feng, Yudan Wu, Wenbo Zhang, Ling Wei, Huiting Che, Le Xue, Yidan Gao, Ji Tao, Shufang Qian, Wenzhao Cao, Jun Zhang, Chengyan Wang, Mei Tian

**Affiliations:** 1 Human Phenome Institute, Fudan University, 825 Zhangheng Road, Pudong New District, Shanghai, 201203 China; 2 Department of Nuclear Medicine and PET Center, The Second Affiliated Hospital of Zhejiang University School of Medicine, Hangzhou, 310009 Zhejiang China; 3 Department of Radiology, Huashan Hospital, State Key Laboratory of Medical Neurobiology, National Center for Neurological Disorders, Fudan University, Shanghai, 200040 China

**Keywords:** Phenomics, Imaging, Genomics, Transcriptomics, Proteomics, Immunomics, Metabolomics

## Abstract

Human phenomics is defined as the comprehensive collection of observable phenotypes and characteristics influenced by a complex interplay among factors at multiple scales. These factors include genes, epigenetics at the microscopic level, organs, microbiome at the mesoscopic level, and diet and environmental exposures at the macroscopic level. “Phenomic imaging” utilizes various imaging techniques to visualize and measure anatomical structures, biological functions, metabolic processes, and biochemical activities across different scales, both in vivo and ex vivo. Unlike conventional medical imaging focused on disease diagnosis, phenomic imaging captures both normal and abnormal traits, facilitating detailed correlations between macro- and micro-phenotypes. This approach plays a crucial role in deciphering phenomes. This review provides an overview of different phenomic imaging modalities and their applications in human phenomics. Additionally, it explores the associations between phenomic imaging and other omics disciplines, including genomics, transcriptomics, proteomics, immunomics, and metabolomics. By integrating phenomic imaging with other omics data, such as genomics and metabolomics, a comprehensive understanding of biological systems can be achieved. This integration paves the way for the development of new therapeutic approaches and diagnostic tools.

## Introduction

Phenomics is a complex and cutting-edge field of science that involves high-dimensional phenotypic data on an organism-wide scale. It is a subject that involves biological, chemical, and physical traits acquired through various approaches including biological sampling, questionnaires, and physical measures (Jin 2021). Since traditional multi-omics methods are insufficient in capturing the spatial aspects of human phenome, recently, molecular/medical imaging technologies emerged as a novel approach for this purpose. Therefore, we propose the term "phenomic imaging" to describe the utilization of imaging approaches for the comprehensive analysis of human phenomics, which encompasses a diverse range of morphological, physiological, functional, and molecular characteristics. The main advantage of phenomic imaging is that it enables the acquisition of non-invasive, repeatable, and spatio-temporal dynamic data, which provides snapshots of dynamic and instant changes in organs of interest (Nagle et al. 2021).

Molecular/medical imaging is primarily used for disease diagnosis, whereas phenomic imaging is used for precise qualitative assessment, localization, and quantitative characterization of phenotypes (Bai et al. 2020). Phenomic imaging captures planar or stereoscopic images of phenotypes, and assists in investigations of the relationship between imaging phenotypes and other biological features, thereby extracting highly representative and qualifiable structural or functional features from high-throughput data (Yu et al. 2022). By combining genomics, transcriptomics, proteomics, immunomics, and metabolomics with phenomic imaging, we are able to visualize complex phenotypic data and explore the correlation between macro- and micro-phenotypes (Schroder et al. 2018; Shui et al. 2020; Wissler et al. 2019).

**Fig. 1:**
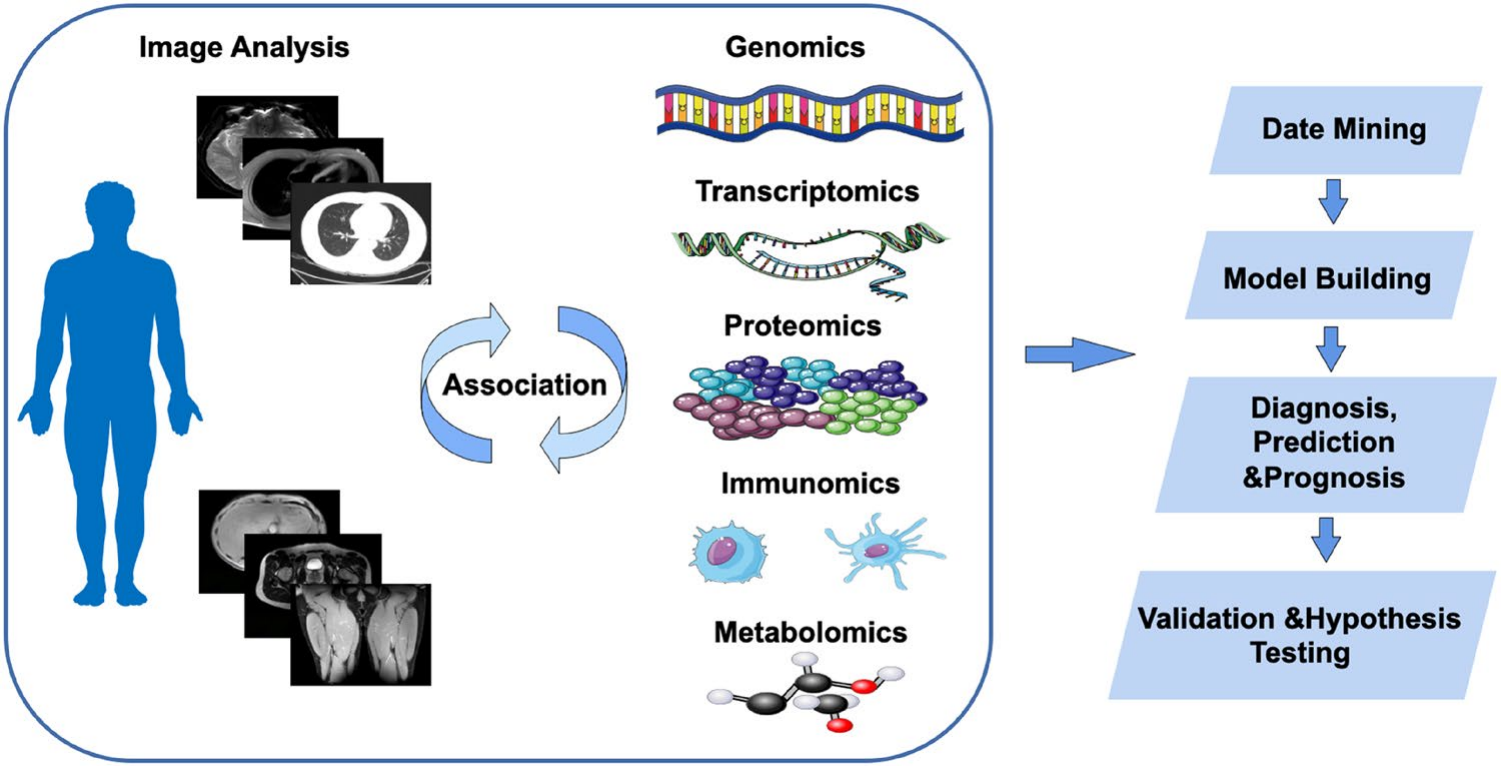
The process for conducting phenomic imaging studies involves three main steps: image analysis, integration of data from other omics, and data analysis. The first step involves analyzing images to extract features related to the traits being studied. The second step involves combining imaging data with data from other omics fields to better understand how the traits are influenced by various factors. Finally, data analysis techniques are used to validate hypotheses about the relationships between the traits and other biological features. Overall, this approach allows researchers to obtain more comprehensive understanding of an individual's phenotype and the underlying biological mechanisms

Phenomic imaging plays a vital role in acquiring accurate and reliable phenotypic data, which is the premise to ensure the accuracy and reliability of biological phenotype analysis. Besides its use in diagnosis, phenomic imaging can also be used for disease susceptibility assessment, prevention, and treatment (Shui et al. 2020; Zhao et al. 2022). With the combination of multi-omics, phenomic imaging allows us to visualize complex phenotypic data and explore the correlation between macro- and micro-phenotypes (Fig. 1). This approach has the potential to uncover new insights and improve our understanding of the complex interplay between the various factors that contribute to an individual's phenotype (Diamanti et al. 2020).

## Phenomic Imaging Modalities

The study of human anatomy has long been a fundamental part for us to understand the human body. However, many observable characteristics of organs and systems have remained enigmatic in relation to microscopic components such as small molecules and genetic materials (Bellis 2021). Technological advances have enabled us to shed light on this mystery by facilitating the depiction of observable features with unprecedented precision. The invention of microscope, for instance, allowed the visualization of microscopic structures, significantly contributing to our understanding of the infinitesimal (Muthumbi et al. 2019). Medical imaging technologies have also played a crucial role in the development of radiomics by providing non-invasive, real-time, and high-resolution images of various organs and tissues both in vivo and in vitro since the 20th century.

**Fig. 2:**
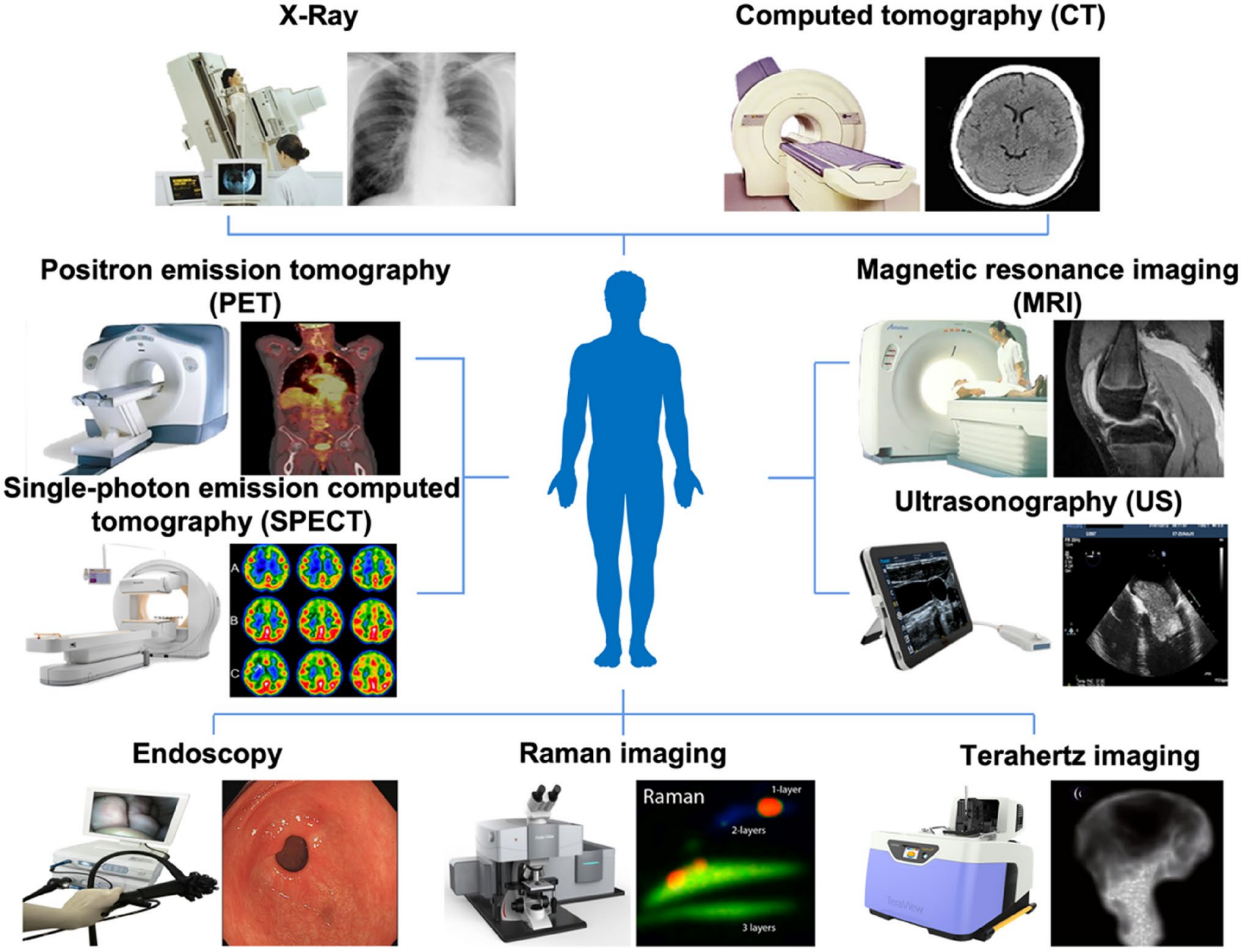
There are several biomedical imaging modalities that are currently available in medical research and clinical settings. These include X-ray, computed tomography (CT), magnetic resonance imaging (MRI), positron emission tomography (PET), single-photon emission computed tomography (SPECT), ultrasonography (US), endoscopy, Raman imaging, and terahertz imaging. Each of these modalities has its own unique strengths and limitations, making them suitable for different types of applications. For example, some modalities are better for visualizing soft tissues, while others are more effective at detecting changes in metabolic activity or molecular structure

Phenomic imaging technologies include X-ray, computed tomography (CT), magnetic resonance imaging (MRI), positron emission tomography (PET), single-photon emission computed tomography (SPECT), ultrasound (US), and other imaging modalities (Fig. 2). Each imaging technique has the potential to reveal unique characteristics of research subjects at different levels, from the molecular level of metabolism and biochemical processes to the cellular level of cell features and the organismal level of different organs (Li et al. 2011).

### X-Ray

There are two types of X-rays used in phenomic imaging: hard X-rays and soft X-rays. Hard X-rays have shorter wavelengths and greater energy, while soft X-rays have longer wavelengths and lower energy. X-ray imaging works by utilizing the radiation characteristics of X-rays, which include penetration, fluorescence, photographic, and ionization effects. Different tissues absorb X-rays at different rates due to differences in their physical densities (Guillou et al. 2022). Diagnostic X-ray imaging has been an essential tool in patient diagnosis and management for over 100 years, evolving significantly over time (Aksoy et al. 2021).

### Computed Tomography (CT)

CT is an imaging modality that uses X-rays to produce three-dimensional (3D) images of internal organs based on reconstructed tomographic measurements taken from different angles. The first CT scanner was invented by Godfrey Hounsfield in 1977 (Goodman and McHugh 1997), and since then, CT equipment has evolved to the fifth generation, with an expanded inspection scope from the brain to the whole body. Contrast-enhanced CT is another type of CT scan that is commonly used to highlight lesions in normal tissue, and materials can be differentiated further by applying different X-ray spectra and analyzing the differences in attenuation (Nagayama et al. 2022). Dual-energy CT is also available, which obtains an additional attenuation measurement with a second X-ray spectrum to allow the differentiation of multiple materials (Hsu et al. 2020). Multi-energy CT or spectral CT can even provide information about the elemental composition of an object by measuring the energy-dependent material-specific X-ray attenuation in three or more distinct energy regimes (McCollough et al. 2015). Photon counting CT has demonstrated great potential in providing high-quality images with reduced radiation dose, making it a promising tool for a wide range of clinical and research applications, including diagnostic imaging, tissue characterization, and material analysis (Si-Mohamed et al. 2022; Willemink et al. 2018).

### Magnetic Resonance Imaging (MRI)

MRI utilizes the nuclear magnetic resonance of atoms in the body's tissue induced by radio waves (Bloch et al. 1946; Purcell et al. 1946). Variations in relaxation time of different tissues generate different signals on MR images. Magnetic resonance hydrography can display the entire liquid pipeline system in the human body without a contrast agent, such as magnetic resonance cholangiopancreatography (MRCP), magnetic resonance urography (MRU), and magnetic resonance myelography (MRM). Magnetic resonance angiography (MRA) uses the liquid flow effect principle to display blood vessel structures in the form of time-of-flight (TOF) (Li et al. 2021). Susceptibility-weighted imaging (SWI) can show the differences in magnetic susceptibility between normal tissues or between normal tissues and lesions. Magnetic resonance spectroscopy (MRS) can detect miniature biochemical differences in normal tissues and lesions because the resonance frequency of ^1^H differs in different compounds (Reynolds et al. 2017). Diffusion-weighted imaging (DWI) and diffusion tensor imaging (DTI) can reflect the diffusion of water molecules in tissues or lesions and their restricted degree. Blood oxygen level-dependent (BOLD) imaging can localize brain function during specific brain activities. Dynamic contrast-enhanced MRI (DCE-MRI) and dynamic susceptibility contrast MRI (DSC-MRI) are both valuable MRI techniques that utilize contrast agents to visualize and assess blood vessels and blood flow in the body (Smith et al. 2012). Chemical exchange saturation transfer (CEST) is a contrast mechanism in MRI that provides distinct advantages for quantitative imaging. It offers valuable insights into various biophysical and biochemical tissue parameters that are closely associated with exchange-coupled magnetization pools (van Zijl et al. 2018). MRI has high image resolution, excellent image contrast, and is free of ionizing radiation, making it ideal for surgical procedures like tumor resection, grading brain/heart infarction, and evaluating inflammation. As a result, it has become an essential tool in clinical medicine and medical research.

### Positron Emission Tomography (PET)

The PET technique was invented in 1975 by Michel Ter-Pogossian, Michael E. Phelps, and Edward J. Hoffman (Ter-Pogossian et al. 1975). PET uses positron-emitting radiotracers to label body metabolites. It is a representative imaging technique that reflects metabolic changes and provides metabolic information to allow general evaluations of patients (Wang et al. 2023). PET/CT and PET/MRI have integrated the advantages of radiology and molecular imaging, providing both anatomical and functional information to permit lesion analysis from a single platform (Zhang et al. 2021). A standard PET system contains only a small portion of the body within its field of view (FOV) despite the systemic tracer injection and presence of the radiotracer throughout the entire body. Therefore, a 200-cm FOV total-body PET scanner has much higher sensitivity than a typical 20-cm FOV scanner. The TOF information provides a more accurate location of the annihilation event, leading to improved signal-to-noise ratio (SNR) in the reconstructed image (Cherry et al. 2018).

### Single-Photon Emission Computed Tomography (SPECT)

SPECT, or single-photon emission computed tomography, is a molecular imaging technique that uses gamma rays emitted from radioisotopes to produce tomographic images of radiotracer distribution (Bajc et al. 2019). It has been widely used in clinical nuclear medicine, nuclear cardiology, and nuclear neurology for several decades. SPECT was first developed for clinical application and has since become commercially available (Hutton 2014). Recently, hybrid SPECT/CT systems have been developed, which combine the functional information of SPECT with the anatomical information of CT to provide more accurate imaging data and pathophysiologic information about diseases (Dickson et al. 2023; Israel et al. 2019). These systems have shown significant clinical value in areas such as oncology (Schmidkonz et al. 2018), neurology (Sood et al. 2021), and cardiology (Scully et al. 2020; van de Burgt et al. 2021).

### Ultrasound (US)

US was first applied to the human body by Karl Theo Dussik to outline the human brain ventricles (Shampo and Kyle 1995). Us or sonography is based on the acoustical impedance of moving sound waves and is used for medical diagnosis. Sonography diagnosis has several modes, including A-mode, B-mode, M-mode, and Doppler mode. A-mode provides the simplest and most original information from echo, a function of depth. B-mode utilizes probes with sensor arrays to construct two-dimensional (2D) information. M-mode records the signal of motion by applying pulse waves, while Doppler mode utilizes the principle of Doppler to record the speed and direction of blood flow (Yao et al. 2017). Ultrasonography is commonly used in various medical specialties such as orthopedics, gynecology and obstetrics, otolaryngology, and urology. It can also be used for angiology, gastroenterology, and colorectal surgery with specific probes (Tessler et al. 2018).

### Others

Various imaging modalities can provide insights into human phenomics. For instance, 3D human body scanning technology can produce digitized information of face and body shape (Pleuss et al. 2019). Endoscopes, such as laryngoscopes and bronchoscopes, can provide real-time views of human respiratory and digestive tract tissues. Ventriculostomy and thoracoscopy can be used to obtain images of the body cavity during surgical operations, contributing to the diagnosis and treatment of mucosal tissue diseases. Raman imaging can provide information about molecular composition and cellular structure, allowing for the differentiation of tumors from normal tissue under surgical or ex vivo conditions (Ramya et al. 2021). Terahertz waves can be used for early caries detection, identification of ex vivo tissue samples, and intraoperative imaging for identifying tumor margins (Amini et al. 2021). Table 1 summarizes the imaging modalities and their basic characteristics.

**Table 1:** Phenomic imaging modalities and basic characters

Medical imaging modality	Imaging source	Advantages	Disadvantages	Application
X-ray imaging	X-ray	High space resolution, easy to operate	Low contrast resolution, tissue overlap, restricted imaging depth	Internal organ diagnosis, contrast examination
CT	X-ray	High contrast resolution, clear anatomy structure, high imaging depth	Low contrast for soft tissue	Internal organ diagnosis, perfusion imaging, angiography
MRI	RF pulses	High contrast for soft tissue, high spatial resolution, high imaging depth, ionizing radiation-free	Distortion, low imaging speed	MRCP, MRU, MRM, MRA, MRS, SWI, DWI, DTI, BOLD, DCE-MRI, DSC-MRI, CEST
PET	γ photon	Quantitative imaging, better imaging quality (compared with SPECT), high imaging depth	Low spatial resolution	Molecular functional imaging, reflects the metabolic information
SPECT	γ photon	Quantitative imaging, more functional imaging probes (compared with SPECT), high imaging depth	Low spatial resolution	Early detection of disease diagnosis, lesion location
US	Ultrasound	High temporal resolution, ionizing radiation-free	Restricted imaging depth, high operator dependence	Widely organ diagnosis, operation-aided imaging, ultrasound intervention
Endoscopes	Photoelectric sensor	Visual information	Information content limited, invasive detection	Laryngoscopy, bronchoscopy, thoracoscopy
Raman imaging	Raman spectra	–	–	Tumor differentiation
Terahertz wave imaging	Terahertz wave	–	–	Early caries detection, tumor margin identification

## Applications

In traditional scientific research, the correlation between macroscopic features and microscopic phenotypes is observed to bring about high accuracy and feasibility, albeit with an inefficient and prolonged verification process, leading to a drop in the bench-to-bedside transition incidence. The human body has numerous measurable phenotypes from micro to macro, and their associations grow exponentially. Hence, phenomenological research aims to expedite the discovery of these connections between macro- and micro-phenotypes to accelerate the transition from scientific research to clinical practice (Jin 2021).

### Imaging Genomics

Imaging genomics is an emerging field that aims to identify the associations between image phenotypes and genomic information using advanced image processing and analysis techniques (Cho et al. 2017). Medical imaging modalities such as CT, MRI, and PET are used to convert the structure and function of human organs or tissues into quantifiable features, which can reflect the underlying molecular and genotypic basis for the tissue of interest (Shin et al. 2016). The ultimate goal of imaging genomics is to develop imaging biomarkers that combine phenotypes and genotypes to predict risk and/or outcome, which can accelerate the progression of precision medicine (Shui et al. 2020).

Imaging genomics can be performed using non-invasive multimodal (X-rays, CT, PET, MRI) or multiparametric (multiple MRI sequences, such as diffusion MRI, perfusion MRI) techniques. In cancer research, imaging genomics offers a more inclusive view of tumors compared to tumor biopsies (Chen et al. 2015). PET-based radiomic features can predict genetic alterations and complement the imaging genomics approach by incorporating anatomical information, thereby strengthening multiparametric prediction models. Moreover, imaging features extracted from PET or PET/CT allow in vivo functional and physiological activity assessment, and provide comprehensive information about tumors non-invasively. The association between imaging features and gene expression can provide valuable insights, such as applying imaging features in predicting oncogene expressions. Gevaert et al. (2012) were the first one to use the imaging genomic approach to identify prognostic imaging biomarkers by correlating imaging features to metagenes and aggregated gene expression patterns in lung cancer. In another study, associated features were incorporated into multiparametric models to predict mutations in specific genes or genetic pathways (Vlachavas et al. 2019).

Several studies have investigated the potential of ^18^F-Fluorodeoxyglucose-positron emission tomography (^18^F-FDG-PET) in assessing *Kirsten rat sarcoma viral oncogene homolog* (*KRAS*) mutations (Chen et al. 2014, 2015; Lovinfosse et al. 2016), but their findings have been inconsistent. While some studies have found significant associations between standard uptake value (SUV) intensity features like SUVmax, SUV histogram features, and volumetric features and *KRAS* mutation (Chen et al. 2014), others have refuted these findings and found no meaningful association between PET parameters and *KRAS* status (Lovinfosse et al. 2016).

Imaging genomics can be used to develop imaging biomarkers as surrogates for genetic testing, a unique advantage of this field (Hu et al. 2017). Many previous studies have attempted to predict various hallmark mutations non-invasively, such as *isocitrate dehydrogenase (IDH)* (Arita et al. 2018; Bisdas et al. 2018), *tumor protein 53 (TP53)* (Hu et al. 2017), *epidermal growth factor receptor (EGFR)* (Akbari et al. 2018; Li et al. 2018b), and *1p/19q* codeletion (Shofty et al. 2018; Zhou et al. 2017). These gene mutations are reliable prognostic biomarkers in glioblastoma and are associated with MRI features. Additional imaging markers predicting clinically relevant gene expression have also been identified, such as *Ki-67* (Li et al. 2017), *alpha thalassemia/mental retardation syndrome X-linked* (*ATRX*) (Li et al. 2018a), *branched-chain amino acid transaminase 1 * (*BCAT1*) (Cho et al. 2017), *platelet-derived growth factor receptor alpha* (*PDGFRA*) (Hu et al. 2017), *phosphatase and tensin homolog deleted on chromosome 10* (*PTEN*) (Zinn et al. 2017), *retinoblastoma 1* (*RB1*) (Hu et al. 2017), and CD3 RNA expression (Narang et al. 2017).

Imaging genomics also enables the correlation of MRI features with clinically available genomic assays to provide prognostic scores for cancer recurrence and guide treatment decisions (Grimm et al. 2017).These investigations shed light on local tumoral environments, which advances our understanding of tumor biology and identifies potential imaging surrogates of molecular subtypes. Additionally, artificial intelligence (AI) can efficiently process a large quantity of medical imaging data, providing a scientific approach to computer-assisted medical diagnosis. AI technologies, such as deep learning, have made significant progress in medical image recognition and genome analysis. AI-assisted cardiac imaging genomics has demonstrated promising results in solving cardiovascular research challenges, such as complex disease classification, clustering, and predictive modeling tasks. These studies aim to identify and characterize genetic variants influencing functional, physiological, and anatomical phenotypes derived from cardiovascular imaging (Bai et al. 2020).

### Imaging Transcriptomics

The combination of transcriptomic and imaging data is referred to as "imaging transcriptomics" (Martins et al. 2021). Transcriptomics involves the study of RNA transcripts, which act as intermediates between DNA and protein. It provides functional context to essential genes and regulatory mechanisms that exhibit selective expression patterns in pathogenesis (Wang et al. 2009). In recent years, researchers have explored the integration of transcriptomic expression profiles with imaging features to non-invasively study the molecular characteristics of various tumor types and predict clinical outcomes (Cruickshank-Quinn et al. 2018; Li et al. 2020).

The combination of structural medical imaging modalities like X-rays, MRI, and CT with transcriptomics has led to significant advances in the treatment of clinical diseases. For instance, a study used MRI to image markers of cerebral small vessel disease and identified 66 candidate genes through transcriptome-wide association analysis, highlighting the role of the cerebrovascular matrix and inflammatory mechanisms in this disease (Persyn et al. 2020). Another study correlated MRI phenotypes with The Cancer Genome Atlas (TCGA) to non-invasively detect key cancer genomic components responsible for cell migration and invasion in glioblastoma (GBM) multiforme (Brisse et al. 2017). In a study of non-small cell lung cancer (NSCLC) patients, imaging transcriptomics signatures based on serum microRNA (miRNA) levels and CT texture features were established to predict objective response rate, overall survival, and progression-free survival, facilitating precision treatment development (Fan et al. 2020). Transcriptomics approaches were also used to study biological pathways associated with lung function measured by CT in chronic obstructive pulmonary disease patients (Cruickshank-Quinn et al. 2018). These findings demonstrate the potential of imaging transcriptomics to advance our understanding of the molecular basis of diseases and improve patient outcomes (Martins et al. 2021).

Combining transcriptomics with medical imaging techniques has opened up new possibilities for non-invasive disease diagnosis, prognosis, and treatment. For example, in a study of GBM multiforme, MRI was used to obtain phenotype data of edema, tumor, and necrosis, which were then correlated with TCGA to identify key cancer genomic components responsible for cell migration and invasion. This approach provided a new diagnostic method for GBM multiforme and revealed molecular correlates of cancer subtypes and cell invasion (Brisse et al. 2017). Similarly, a study of NSCLC used serum miRNA levels and CT texture features to establish imaging transcriptomics signatures that predicted the objective response rate (ORR), overall survival (OS), and progression-free survival (PFS) in patients. These findings could potentially lead to the development of precision treatment for NSCLC (Fan et al. 2020).

Other studies have focused on the relationship between medical imaging and tumor biology. In one study of low–intermediate-risk prostate cancer, MRI results were found to be associated with the biological features of aggressive prostate cancer, potentially improving treatment options for patients (Houlahan et al. 2019). For clear cell renal cell carcinoma (ccRCC), a study correlated quantitative data obtained from multiphase CT with the expression of selected miRNA to reveal that the quantitative value of CT examination was related to *miR*-*21*-*5p*, suggesting that patients with ccRCC could benefit from non-invasive texture parameter evaluation of biopsy results (Marigliano et al. 2019). These examples highlight the potential for imaging transcriptomics to provide a more comprehensive understanding of disease mechanisms and improve patient outcomes.

### Imaging Proteomics

Imaging proteomics is a research field that aims to analyze the complex relationship between proteomics, imaging biomarkers, and extracted imaging features. Proteomics research involves the study of the proteome, including the identification of protein targets and abnormal signaling pathways, and it mainly utilizes mass spectrometry. On the other hand, imaging modalities such as PET, MRI, and CT are used to extract structural and functional characteristics of biological tissues, organs, and even the entire body. By analyzing the relationship between targets and image features, potential disease correlations and biomarkers can be identified (Schroder et al. 2018).

Functional molecular imaging techniques, such as PET and SPECT, and non-radionuclide-based methods, such as fMRI, have been widely used to develop potential biomarkers for the diagnosis, monitoring, and prognosis of Alzheimer's disease (AD) (Wang et al. 2021). These techniques can detect behavioral responses in the neocortex by capturing the unique compositional characteristics of the postsynaptic proteome in each brain region and the extensive glucose metabolism. Blood protein biomarkers have also been studied extensively, and they can detect many pathobiological changes of various categories, including metabolic, neuronal, axonal, glial, inflammatory, and vascular levels (Park et al. 2019; Westwood et al. 2016).

PET can quantify cerebral amyloid deposition, contributing to altered brain pathophysiology in AD by targeting and labeling related proteins. Using PET quantification that assesses the proteomic differences in the serum composition among AD patients, patients with cognitive impairment, and healthy individuals, numerous proteins, such as proprotein convertase subtilisin/kexin type 9 (PCSK9), coagulation factor XIII A1 subunit (F13A1), and donation after cardiac death (DCD), have been identified as potential markers for amyloid deposition. Such a breakthrough is a simple, non-invasive approach to diagnosing AD severity or even predicting AD onset (Park et al. 2019; Westwood et al. 2016).

Imaging proteomics can improve clinical diagnosis and help promote our understanding of specific disease mechanisms. For example, PET imaging combined with proteomic analysis has been used to measure the heart's glucose uptake capacity during cardiopulmonary activity assessment. Guo et al. found that panax quinquefolium saponins (PQS) can significantly improve cardiac function and glucose utilization, they also showed that PQS blocks neovascularization by affecting the expression level of protein kinase C delta (PRKCD), a target of PQS (Guo et al. 2018). In another study, an analysis was performed using the rubidium-82 PET imaging results of the cardiac function and myocardial blood flow reserve for 97 patients. This analysis led to the discovery of new potential targets within the coronary arteries, including galectin-4 (Gal4), growth differentiation factor 15 (GDF15), tissue-type plasminogen activator (tPA), and von Willebrand factor (vWF), demonstrating a correlation between protein markers and endothelial-independent coronary microvascular dysfunction (Schroder et al. 2018).

PET imaging is one of the first techniques to predict the response to neoadjuvant therapy in esophagogastric cancer. ^18^F-Fluorothymidine (^18^F-FLT), a marker of tumor cell proliferation, is useful in detecting locally advanced gastric cancer, which is more sensitive than ^18^F-FDG-PET (Herrmann et al. 2007). Future correlation studies between PET imaging and proteomics may further promote the development of personalized gastric cancer treatment. In conclusion, functional molecular imaging techniques and imaging proteomics have shown great promise in identifying specific biomarkers for diagnosing, monitoring, and predicting the prognosis of various diseases. The identification of specific biomarkers can also elucidate treatment responses or drug resistance on the molecular, protein, or even metabolic level.

The advancement of MRI, US, and CT imaging techniques has enabled better understanding of the cardiopulmonary structure and functions (Hemnes et al. 2020). The combination of imaging techniques and proteomic analysis has allowed for the identification of abnormalities in axonal or vascular damage markers, metabolic anomalies, and mechanisms underlying brain injury or atrophy (Herman et al. 2018; Wright et al. 2019).Proteomic analysis in conjunction with postoperative MRI imaging has shown a correlation between levels of matrix metalloproteinases (MMPs) and tissue inhibitors of metalloproteinases (TIMPs) with clinical outcomes, ventricular dysfunction, and pulmonary artery remodeling (Schafer et al. 2021). In addition, imaging techniques like MRI and CT can provide information about the body's fat compositions, and by combining imaging results with proteome analysis, potential agents like Cajanolactone A have been identified for preventing postmenopausal obesity and fatty liver (Luo et al. 2020). Machine learning has been utilized to non-alcoholic fatty liver disease (NAFLD) and identify biological players in liver fat accumulation (Atabaki-Pasdar et al. 2020). The combination of transcriptomics, proteomics, copy number alterations, and radiomic image features has characterized primary tumors in patients with epithelial ovarian cancer, and correlations between proteins and CT-based imaging features have shown possible associations between tumor heterogeneity and protein abundance (Lu et al. 2019). Furthermore, the investigation of drugs or target ligands is continuously being explored to support the future diagnosis of diseases through imaging targets (Rothlisberger et al. 2017).

### Imaging Immunomics

Imaging immunomics is a rapidly growing field that aims to identify imaging biomarkers to support clinical decision-making and enhance the understanding of disease biology, allowing for assessment of the physiological state and changes throughout the disease progression and therapeutic sequence (Limkin et al. 2017). Various imaging techniques are used for evaluation, including radiomic methods with CT, MRI, PET, near-infrared fluorescence (NIRF), and radiolabeled small molecules, antibodies, and fragments to image immune status, tumor microenvironment, and changes throughout therapy (Shields et al. 2018). Labeled monoclonal antibodies (mAbs) represent a promising tool for immuno-PET theranostic approaches, offering a non-invasive method to assess in vivo target expression and distribution (Wei et al. 2020). Immuno-PET combines the excellent targeting specificity of mAbs with the superior sensitivity and resolution of PET, and represents a paradigm shift for molecular imaging modalities (Knowles and Wu 2012).

The field of imaging immunomics has made significant progress in recent years, with various imaging methods being employed to evaluate immune status, tumor microenvironment, and changes throughout therapy (Keyaerts et al. 2016; Pandit-Taskar et al. 2016). Immuno-PET, in particular, has emerged as a promising tool for molecular imaging, allowing for the non-invasive assessment of in vivo target expression and distribution (Nagle et al. 2021). Immuno-PET has been successfully used to image chronic cardiac rejection and various cancers, and there is much interest in using it to target antigens of pathogenic bacteria and viruses. A predictive radiomics model has been developed to target immune components and patient response to immune checkpoint therapy (Sun et al. 2018). Anti-CD8 immuno-PET has proven to be a sensitive tool for detecting changes in systemic and tumor-infiltrating CD8 T-cell expression, while immuno-PET imaging of inducible T-cell costimulators (ICOS), enables the sensitive and specific detection of activated T cells (Xiao et al. 2020). Immuno-PET studies of programmed death-ligand 1(PD-L1) have also shown promise in predicting the effectiveness of programmed cell death 1(PD-1)/PD-L1 checkpoint blockade therapy (Vento et al. 2019).

The use of imaging immunomics can be expanded to the detection and assessment of inflammatory diseases. PET imaging targeting microglia and lipopolysaccharide has been studied for evaluating medications for cognitive deficits associated with neuroinflammatory dysfunction (Wissler et al. 2019). Another study utilized PET imaging targeting c-reactive protein to investigate the relationship between peripheral inflammation, blood–brain barrier permeability, and behavioral symptoms like depression (Turkheimer et al. 2021). These imaging methods have provided new insights into understanding how inflammation affects brain homeostasis and how it could be manipulated to treat cognitive and behavioral symptoms associated with inflammatory diseases.

Recent studies have highlighted the potential of molecular imaging in evaluating the effectiveness of targeted drugs for immune-mediated inflammatory lung diseases. Rituximab, a mAb that targets CD20-expressing B lymphocytes, has shown promising results in treating such diseases. In addition, antibody-guided PET/MRI has emerged as a state-of-the-art approach for the molecular imaging of invasive pulmonary aspergillosis, offering new insights into the underlying mechanisms of targeted drug imaging (Thornton 2018).

There are also exciting developments in the use of molecular imaging for non-invasive imaging of lung adenocarcinoma. For instance, mAb109 is a new platform for developing novel imaging agents for this type of cancer. Studies have shown that ^64^Cu-NOTA-mAb109/Cy5.5-mAb109 exhibits high specificity and sensitivity for A549 tumors and human lung adenocarcinoma tissues (Zhu et al. 2020). Furthermore, a CD146-targeting probe has been developed as a PET and NIRF imaging agent for hepatocellular carcinoma. This probe showed high affinity and specificity for CD146-expressing liver malignancies, and correlated tracer uptake with in situ CD146 expression, indicating its potential for early detection, prognostication, and image-guided surgical resection of liver tumors (Hernandez et al. 2016).

Immuno-PET has the potential to revolutionize personalized medicine by enabling better patient selection for antibody-based therapies and improving drug development. Despite being relatively expensive, it has been shown in several large multi-center randomized clinical trials to be a valuable primary patient screening method that can help avoid unnecessary procedures (Zhu et al. 2020). By providing accurate diagnoses and assisting physicians in optimizing therapeutic decisions, immuno-PET can contribute to improving clinical practice and personalized medicine.

### Imaging Metabolomics

Metabolomics provides a comprehensive view of the metabolic network in a certain disease condition and can help identify important biomarkers or therapeutic targets. As metabolite levels are highly responsive to changes in tissue biology, metabolomics can differentiate between tumor and benign tissue or tumor subtype-specific features, including those associated with disease stage (Zaimenko et al. 2017). Multiparametric MRI can also report on aspects of tumor biology that influence metabolism, which is important in understanding the biological heterogeneity of solid tumors. Techniques that image biological heterogeneity may help guide tissue sampling for metabolomics and related studies (Glaab et al. 2019). Combining the information from metabolomics and imaging techniques can provide a more complete understanding of tumor biology and guide personalized treatment decisions.

Metabolomics, when combined with PET imaging, can provide insights into metabolic changes in various diseases. For instance, C–C motif chemokine ligand 5 (CCL5) was found to be essential in maintaining hippocampal integrity and energy metabolism, as revealed by metabolomics and FDG-PET analysis (Ajoy et al. 2021). The combination of ^18^F-FDG, ^18^F-fluorodihydroxyphenylalanine-PET (^18^F-DOPA-PET), and metabolomics data improved the accuracy of Parkinson's disease diagnosis (Glaab et al. 2019). In pulmonary arterial hypertension, metabolic reprogramming, including upregulated glutamine and altered glycine and choline metabolism, can be detected through PET imaging in mouse models (Izquierdo-Garcia et al. 2018). Radiation-induced metabolism shifts in hepatocellular carcinoma lesions, involving increased glycolysis and impaired gluconeogenesis, were revealed using FDG-PET and metabolomics (Chung et al. 2021). In type 2 diabetes and insulin resistance, correlations were found among PET/MRI parameters, metabolites from metabolomics profiling of subcutaneous adipose tissue and plasma, which provided new insights into the research area (Diamanti et al. 2020). High expression of thyroid hormone-binding protein μ-crystallin (CRYM) was associated with low choline uptake in ^18^F-fluoromethylcholine (FMC) PET/MRT studies of prostate cancer patients, suggesting that CRYM expression may be a potential biomarker for prostate cancer (PCa) diagnosis and prognosis (Aksoy et al. 2021).

Collaboration between MRI and metabolomics has been observed in various research fields. One study investigating transient ischemic attack (TIA) patients found specific metabolomic profiles related to MR-DWI features, with 11 common molecules identified, such as creatinine and lysophosphatidic acid (Purroy et al. 2016). Similarly, 41 circulating metabolites were found to be significant in the early diagnosis of ischemic stroke, including DWI-positive lesions (Tiedt et al. 2020). In cardiovascular research, metabolites such as serine, citrate, and valine were found to be associated with left atrial function based on cardiovascular magnetic resonance (CMR) imaging (Koh et al. 2018). In patients with ST-segment-elevation myocardial infarction (STEMI), succinic acid was found to be significantly increased in the coronary sinus blood, and its release was associated with the degree of myocardial ischemia, as evaluated by CMR imaging (Kohlhauer et al. 2018). In NAFLD, a cross-sectional analysis identified 10 serum metabolites with high diagnostic accuracy for advanced fibrosis, as shown by MR elastography (Caussy et al. 2019). Additionally, a study combining multi-dimensional data from various omics imaging techniques, including metabolic parameters from NMR spectroscopy and mass spectrometry, MRS, and DTI, demonstrated the importance of the glycine-serine-threonine axis in intervertebral disc degeneration pathology (Wu et al. 2021).

## Challenges and Future Perspectives

In recent years, there has been growing interest in the combination of phenomic imaging modalities with omics studies, which involves high-throughput data extraction of quantitative features from images and convert them into high-dimensional data. This integration has been widely investigated in various fields, including imaging genomics, transcriptomics, proteomics, immunomics, and metabolomics. Phenomic imaging studies involve in-depth analysis of phenotypic data to explore the internal causal relationship among genes, phenotypes, and the environment, offering promising perspectives for future research (Marcu et al. 2019; Shui et al. 2020).

However, phenomics is a relatively new discipline, and its rapid development has faced some challenges. Individual omics data's heterogeneity, temporal dependency, sparsity, and irregularity limit the full utilization of biomedical information, leading to further problems and challenges such as overfitting and the necessity of intensive computational resources for analysis of a large number of features. To address these issues, there is a need for the development of high-throughput techniques, as well as the advancement of AI technologies to assist in data analysis and phenomic imaging. Despite this, key considerations that need improvement include data volume, data quality, temporality, domain complexity, and interpretability. With continued advancements, phenomic imaging can help us appreciate the complex interactions between perceived phenotypes and underlying microscopic features, leading to a better understanding of the relationship between genes, environment, and phenotype (Arita et al. 2018; Bisdas et al. 2018; Shui et al. 2020).

Phenomic imaging, along with multi-omics, is rapidly advancing our understanding of disease pathogenesis and mechanism, leading to the development of targeted drugs and precise disease treatment. The integration of data from multiple sources and the use of AI tools have the potential to enable personalized management in clinical practice and herald an era of precision medicine. However, there are still challenges that need to be addressed, including feature enrichment, federated interference, model optimization, standardization, integration, and data sharing. Large-scale prospective studies with high-quality data acquisition are needed to improve the reproducibility of phenomic imaging studies. Additionally, the time component of phenomic imaging should be considered to provide additional information on the evolution of features.

Transpathology, which considers the integration of multi-scale pathological data, has the potential to advance the understanding of pathophysiological mechanisms. Similarly, recognizing phenomic imaging as a distinct branch of phenomics could accelerate progress in various areas, including scientific research, industry, and society as a whole. By combining phenomic imaging with other omics data, such as genomics and metabolomics, researchers can gain a more comprehensive understanding of biological systems, potentially leading to the development of new therapies and diagnostic tools. However, more research is needed to fully realize the potential of this emerging field, including standardization and data sharing efforts, as well as large-scale prospective studies.

## Conclusion

In conclusion, "Phenomic Imaging" exemplifies an advanced and interdisciplinary approach that utilizes diverse imaging techniques to comprehensively capture observable phenotypes and characteristics influenced by complex interactions at various biological scales. By integrating genetic information, epigenetics at the microscopic level, organ functionality, microbiome dynamics at the mesoscopic level, and the impact of diet and environmental exposures at the macroscopic level, phenomic imaging provides invaluable insights into the intricacies of human phenomes.

This review offers a comprehensive overview of diverse phenomic imaging modalities and their applications in the field of human phenomics. It is evident that phenomic imaging surpasses traditional medical imaging, not only facilitating disease diagnosis but also characterizing both normal and abnormal traits, thereby enabling a detailed correlation between macro- and micro-phenotypes.

Moreover, this approach reveals novel perspectives for understanding human health and disease, visualizing and quantifying anatomical structures, biological functions, metabolic processes, and biochemical activities. Such diverse information acquisition enhances the understanding of phenotypic variations.

## Data Availability

Not applicable.

## Abbreviations

ATRX: Alpha thalassemia/mental retardation syndrome X-linked
AI: Artificial intelligence
AD: Alzheimer's disease
BOLD: Blood oxygen level dependent
BCAT1: Branched-chain amino acid transaminase 1
CCL5: C–C motif chemokine ligand 5
ccRCC: Clear cell renal cell carcinoma
CMR: Cardiovascular magnetic resonance
CT: Computed tomography
F13A1: Coagulation factor XIII A1 subunit
CEST: Chemical exchange saturation transfer
DWI: Diffusion-weighted imaging
DTI: Diffusion tensor imaging
DCD: Donation after cardiac death
DCE-MRI: Dynamic contrast-enhanced magnetic resonance imaging
DSC-MRI: Dynamic susceptibility contrast magnetic resonance imaging
EGFR: Epidermal growth factor receptor
FMC: Fluoromethylcholine
FOV: Field of view
^18^F-FLT: ^18^F-Fluorothymidine
^18^F-FDG-PET: ^18^F-Fluorodeoxyglucose-positron emission tomography
^18^F-DOPA-PET: ^18^F-Fluorodihydroxyphenylalanine-positron emission tomography
Gal4: Galectin-4
GDF15: Growth differentiation factor 15
IDH: Isocitrate dehydrogenase
immuno-PET: Immuno-positron emission tomography
ICOS: Inducible T-cell costimulators
KRAS: Kirsten rat sarcoma viral oncogene homolog
MRI: Magnetic resonance imaging
MRCP: Magnetic resonance cholangiopancreatography
MRU: Magnetic resonance urography
MRM: Magnetic resonance myelography
MRA: Magnetic resonance angiography
MRS: Magnetic resonance spectroscopy
MMPs: Matrix metalloproteinases
mAbs: Monoclonal antibodies
NAFLD: Non-alcoholic fatty liver disease
NSCLC: Non-small cell lung cancer
NIRF: Near-infrared fluorescence
ORR: Objective response rate
OS: Overall survival
PET: Positron emission tomography
PDGFRA: Platelet-derived growth factor receptor alpha
PFS: Progression-free survival
PQS: Panax quinquefolium saponins
PRKCD: Protein kinase C delta
PD-1: Programmed cell death 1
PD-L1: Programmed death-ligand 1
PCSK9: Proprotein convertase subtilisin/kexin type 9
RB1: Retinoblastoma 1
STEMI: ST-segment-elevation myocardial infarction
SPECT: Single-photon emission computed tomography
SWI: Susceptibility-weighted imaging
SUV: Standard uptake value
TOF: Time-of-flight
TP53: Tumor protein 53
PTEN: Phosphatase and tensin homolog deleted on chromosome 10
TCGA: The Cancer Genome Atlas
tPA: Tissue-type plasminogen activator
TIMPs: Tissue inhibitors of metalloproteinases
CRYM: Thyroid hormone-binding protein μ-crystallin
TIA: Transient ischemic attack
US: Ultrasonography
vWF: Von Willebrand factor
